# Design of a generic CRISPR-Cas9 approach using the same sgRNA to perform gene editing at distinct loci

**DOI:** 10.1186/s12896-019-0509-7

**Published:** 2019-03-20

**Authors:** Soumaya Najah, Corinne Saulnier, Jean-Luc Pernodet, Stéphanie Bury-Moné

**Affiliations:** Institute for Integrative Biology of the Cell (I2BC), CEA, CNRS, Université Paris-Sud, Université Paris-Saclay, Gif-Sur-Yvette, France

**Keywords:** CRISPR-Cas9, Essential gene, Multicopy gene, *Streptomyces*, Foreign DNA, Bait DNA, Xenogeneic silencers, Nucleoid-associated proteins, Lsr2, Generic tool

## Abstract

**Background:**

The CRISPR/Cas (clustered regularly interspaced short palindromic repeat and CRISPR-associated nucleases) based technologies have revolutionized genome engineering. While their use for prokaryotic genome editing is expanding, some limitations remain such as possible off-target effects and design constraints. These are compounded when performing systematic genome editing at distinct loci or when targeting repeated sequences (e.g. multicopy genes or mobile genetic elements). To overcome these limitations, we designed an approach using the same sgRNA and CRISPR-Cas9 system to independently perform gene editing at different loci.

**Results:**

We developed a two-step procedure based on the introduction by homologous recombination of ‘bait’ DNA at the vicinity of a gene copy of interest before inducing CRISPR-Cas9 activity. The introduction of a genetic tool encoding a CRISPR-Cas9 complex targeting this ‘bait’ DNA induces a double strand break near the copy of interest. Its repair by homologous recombination can lead either to reversion or gene copy-specific editing. The relative frequencies of these events are linked to the impact of gene editing on cell fitness. In our study, we used this technology to successfully delete the native copies of two xenogeneic silencers *lsr2* paralogs in *Streptomyces ambofaciens*. We observed that one of these paralogs is a candidate-essential gene since its native locus can be deleted only in the presence of an extra copy.

**Conclusion:**

By targeting ‘bait’ DNA, we designed a ‘generic’ CRISPR-Cas9 toolkit that can be used to edit different loci. The differential action of this CRISPR-Cas9 system is exclusively based on the specific recombination between regions surrounding the gene copy of interest. This approach is suitable to edit multicopy genes. One such particular example corresponds to the mutagenesis of candidate-essential genes that requires the presence of an extra copy of the gene before gene disruption. This opens new insights to explore gene essentiality in bacteria and to limit off-target effects during systematic CRISPR-Cas9 based approaches.

**Electronic supplementary material:**

The online version of this article (10.1186/s12896-019-0509-7) contains supplementary material, which is available to authorized users.

## Background

The CRISPR/Cas (clustered regularly interspaced short palindromic repeat and CRISPR-associated nucleases) systems are widespread in prokaryotes, where they naturally confer an acquired and heritable immunity against viruses and foreign nucleic acids [1–3]. The biotechnological applications of CRISPR-Cas have opened new avenues in the fields of genetics, synthetic biology and functional genomics. Notably, these technologies have revolutionized the way mutations can be introduced in a huge diversity of genomes from all kingdoms of life, bacteria [4], eukaryotes [5] and more recently archaea [6].

Genome editing corresponds to the introduction of a desired change into a genome. Class 2 *Streptococcus pyogenes* Type II CRISPR-Cas9 system is currently predominantly used to perform genome editing. It is based on the activity of Cas9, an RNA-guided endonuclease, which introduces double stranded DNA breaks following sgRNA (single guide RNA):genomic DNA base-paring rules [5]. The sgRNA corresponds to the artificial fusion of a crRNA (CRISPR RNA) specifically recognizing the DNA target sequence and a tracrRNA (trans-activating CRISPR RNA). This system can be used as molecular scissors to generate programmable targeted cuts within genomes. These breaks are repaired by the endogenous cellular DNA repair machineries. In prokaryotes the non-homologous end-joining (NHEJ) pathway seems less prevalent than in eukaryotes [4], with certain CRISPR systems even inhibiting this pathway [7]. Therefore, the CRISPR-Cas9 induced DNA damage is mainly repaired by homologous recombination in replicating bacteria. This favors genome editing-strategies based on the introduction of a judiciously designed template for “homology-directed repair” (HDR). This way, genome editing can allow researchers to insert, remove or replace genes in the context of various experimental designs such as gene deletion, site-specific mutagenesis, gene tagging, or reporter gene insertion [4, 8].

These genome-editing technologies have been applied with success in various bacteria of which some are rather recalcitrant to genetic manipulation such as *Streptomyces* [9–14]. These multicellular bacteria possess an extremely GC-rich linear genome with terminal inverted repeats. They are among the main producers of pharmacologically active and industrially relevant natural products [15]. The genetic engineering of *Streptomyces* therefore represents a great challenge to discover new bioactive components and to improve the production of secondary / specialized metabolites of interest.

Several approaches have been developed to perform targeted mutagenesis in these bacteria. These are based on the replacement of the native locus of interest by a deleted and/or mutated version following two events of homologous recombination with a template DNA. The first recombination event is generally easy to select via the integration of a suicide plasmid harbouring a selection marker. However, the selection of the second recombination event is more complicated. It requires a labour-intensive screening to sort clones that have eliminated the plasmid sequences and therefore lost the selection marker. Some strategies have been developed for dominance selection of the second recombination event in *Streptomyces* using counterselectable markers such as: *rpsL* (encoding the ribosomal protein S12) that confers streptomycin sensitivity in streptomycin resistance background [16], *glkA* encoding a glucokinase conferring sensitivity to 2-deoxyglucose [17], or *codA* encoding a cytosine deaminase which converts 5-fluorocytosine into 5-fluorouracil, a highly toxic compound [18]. These procedures require specific host backgrounds, which limits their extensive use. The introduction of DNA damages by I-*Sce*I meganuclease [19] or CRISPR-Cas9 systems [9–14] constitutes an additional and efficient positive selection marker that has allowed the development of powerful tools to manipulate *Streptomyces* genomes.

Despite the great achievement of CRISPR-based technologies in many organisms, a number of limitations remain such as: i) possible toxicity or drawbacks associated with off-targets, ii) design constraints linked to the requirement at the target site of the consensus PAM sequence (depending on the Cas9 version used to perform gene editing), and/or iii) design constraints for avoiding sgRNA pairing at several genomic loci [4]. This latter aspect imposes the implementation of specific strategies when targeting repeated sequences (e.g. multicopy genes or mobile genetic elements). One particular example of such a case is represented by the mutagenesis of candidate-essential genes that requires the presence of an extra copy of the gene before gene disruption.

To circumvent these limitations, we developed a two-step procedure based on the introduction of foreign DNA (further referred as ‘bait’ DNA) at the vicinity of a gene copy of interest before inducing CRISPR-Cas9 activity. We applied this technology to the editing of two paralogs encoding Lsr2 proteins in *Streptomyces ambofaciens*.

Lsr2 proteins are small nucleoid-associated proteins (NAPs) exclusively and ubiquitously present in Actinobacteria, being therefore a ‘signature’ of this bacterial order [20]. They constitute a particular class of xenogeneic silencers (XS) that may repress the expression of horizontally-acquired DNA as H-NS does in Proteobacteria [21, 22]. Lsr2 encoding gene is essential in *Mycobacterium tuberculosis* [23–25]. Moreover, all genomes of *Streptomyces* harbor at least two *lsr2* paralogs [20]. In *S. ambofaciens* these genes are located close to the origin of replication, a region enriched in essential genes [26]. Altogether these observations prompted us to apply a CRISPR-targeting ‘bait’ DNA approach to explore the possible essentiality of *lsr2* paralogs in *S. ambofaciens*.

## Results

### Design of the CRISPR-Cas9 approach targeting ‘bait’ DNA

To address the possible essentiality of the two XS paralogs in *S. ambofaciens*, an extra copy of these genes was integrated at the *att*B site of PhiC31 phage using pSET152 vector (Additional file 1: Table S1).

**Fig. 1:**
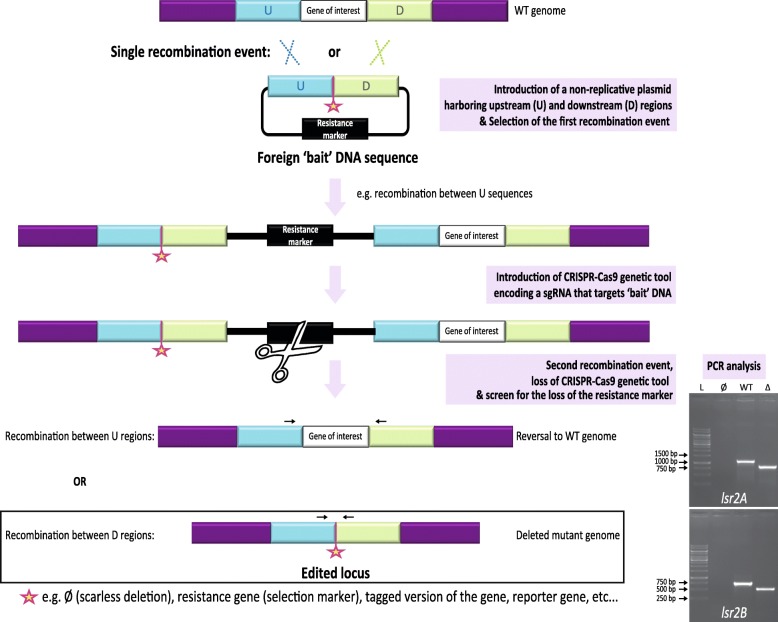
Main stages of the CRISPR-Cas9 targeting ‘bait’ DNA approach to perform gene-copy specific editing. The approach is based on the specific insertion by homologous recombination of a selection marker and a foreign DNA harboring the upstream (‘U’) and downstream (‘D’) regions surrounding the copy of interest. The star represents the various possible designs of the model DNA used to repair the lesion by homologous recombination. For clarity reasons, the scheme only represents a first recombination event occurring between U sequences but the homologous recombination could also take place between D sequences. Thereafter a genetic tool encoding Cas9 and a sgRNA specifically directed against foreign ‘bait’ DNA is introduced transitorily in the recombinant strain. The double strand break induced by this complex can be repaired by a second recombination event between either the U or D sequences, resulting in reversion or in the copy-specific editing, respectively. In absence of a second selection marker at the star position, the distinction between both situations can be achieved by PCR (arrows represent primer positions). The pictures show representative results of PCR performed without template (“Ø”), on the native genome (“WT”) or on the genome of scarless deleted mutants (“Δ”) obtained during this study. The PCR product expected sizes were 1106 pb and 815 pb for native and deleted *lsr2A* loci, and 729 pb and 481 pb for native and deleted *lsr2B* loci, respectively (see Method section for further details). The PCR product expected sizes for native and deleted loci are indicated (“L”: GeneRuler™1 kb DNA ladder, Thermo Scientific)

Thereafter the strains contained 2 identical copies of *lsr2A* (SAM23877-RS19855) or *lsr2B* (SAM23877-RS17060) genes within their genomes. To specifically edit the native locus, we developed an approach based on the introduction of ‘bait’ DNA in the vicinity of the target gene. This was achieved by introducing a non-replicative plasmid harboring the upstream and downstream regions that surround the copy of interest within the native genome (Fig. 1). This ‘bait’ DNA was inserted specifically at the chromosome following a single recombination event possibly occurring between upstream or downstream regions. This single recombination event can be easily selected by introducing a selection marker within the ‘bait’ DNA, the hygromycin resistance in this study. The efficiency of this first stage largely relies on homologous recombination frequency. This may vary notably depending on the species, the length of the homologous regions and the location of the recombination site within the genome [27–29].

We designed a sgRNA targeting this selection marker using CRISPRy-web site analysis (https://crispy.secondarymetabolites.org/#/input) [30] and Cas-OFFinder (http://www.rgenome.net/cas-offinder/) [31] to select a 20-nucleotide target region that is predicted to have no off-targets within *S. ambofaciens* genome. We took advantage of the high GC-content of *S. ambofaciens* genome (circa 72%) to design a sequence with a low GC-content (40%) further minimizing the risk to have off-targets. The introduction of a genetic tool encoding a CRISPR-Cas9 complex targeting this ‘bait’ DNA leaves the second copy of the gene intact whereas it induces a double strand break at the vicinity of the copy of interest (Fig. 1).

This break is then repaired by the cellular machinery. In bacteria the NHEJ pathway is less prevalent than in eukaryotes [4]. If NHEJ-like genes can be involved in genome plasticity and resistance to DNA damage of *S. ambofaciens* [32, 33], the efficient repair of Cas9-induced damages by the NHEJ pathway requires the overexpression of a heterologous DNA ligase D in this bacteria [11]. Therefore in *S. ambofaciens* harboring repeated (upstream and downstream) sequences near the target gene, the CRISPR-Cas9 induced double-strand DNA break stimulates the occurring of a second recombination event between either the upstream or downstream repeated sequences (Fig. 1). In both cases the plasmid sequences are lost, allowing the screening of hygromycin-sensitive clones to identify the clones in which the CRISPR-Cas9 system induced this second recombination event. In this study, we screened at least 30 clones per strain and observed that they were all hygromycin-sensitive after the induction of Cas9 expression. This confirms that HDR is the predominant pathway involved in DNA repair in *Streptomyces* harboring repeated sequences near the double-strand break.

### The scarless deletion of *lsr2* paralogs in *Streptomyces ambofaciens*

Depending on the sequence present between upstream and downstream regions within the ‘bait’ DNA, this CRISPR-Cas9 approach potentially allows gene deletion, replacement or insertion. In our case, upstream and downstream sequences were immediately adjacent so that gene-editing leads to scarless deletion of the copy of interest (Fig. 1). By avoiding the insertion of promoter and antibiotic resistance encoding sequences, scarless deletion is thought to minimize the impact of the deletion on the rest of the genome. This limits biases on mutant phenotype. Since CRISPR-Cas9 action is only required transitorily, we used a thermosensitive plasmid encoding the sgRNA and an inducible Cas9. This transitory expression minimizes the toxicity and potential off-target effects of CRISPR-Cas9 system.

**Fig. 2:**
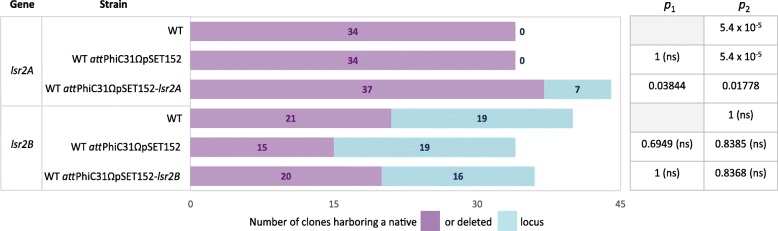
Proportion of native gene copy-deleted clones obtained after the CRISPR-Cas9 approach targeting ‘bait’ DNA. A CRISPR-Cas9 approach targeting ‘bait’ DNA was performed to delete *lsr2A* or *lsr2B* paralogs in *S. ambofaciens* WT or related strains containing an extracopy of *lsr2* paralog (WT *att*PhiC31ΩpSET152-*lsr2X*, *X* being either *A* or *B*). The WT strain containing an empty vector (WT *att*PhiC31ΩpSET152) was used as a control. The number of hygromycin-sensitive clones (i.e. having lost the hygromycin resistance gene after CRISPR-Cas9 targeting) harboring a native or a deleted *lsr2A* or *lsr2B* gene was determined by PCR analysis. The *p*_1_ value represents the *p* value obtained from a Fisher’s Exact test for count data comparing the frequencies of deletion to the WT condition (for a given *lsr2* paralog CRISPR-Cas9 approach). The *p*_2_ value represents the *p* value from the same test performed to compare the frequencies of deletion to a theoretical frequency of 0.5 (using the same total effective as reference). Abbreviation: ns = not statistically significant

To distinguish between the reversal to the parental genome and the copy-specific deletion resulting from the second recombination event, we analyzed by PCR the genetic organization of the target locus in strains containing or lacking an extra copy of *lsr2A* and *lsr2B* integrated at PhiC31 *att*B site. We observed that whereas the frequencies of deletion of the native locus were not statistically different between strains harboring or lacking an extra copy of *lsr2B* (Fig. 2), the deletion of the native *lsr2A* locus was obtained only in the strain containing an integrated copy of *lsr2A* at PhiC31 *attB* site. Since we used the same sgRNA to perform *lsr2A* and *lsr2B* editing, we can exclude a difference in target recognition. These results strongly suggest that *lsr2A* is potentially an essential gene.

Finally, this approach allows the monitoring of the relative proportion of reversal vs. deletion events in different genetic backgrounds. In our case, we observed that the deletion of *lsr2B* was as frequent as the reversal to the WT genotype, regardless of the presence of an extra copy (Fig. 2). Conversely, the deletion of *lsr2A* was statistically less frequent (*p* value of 0.01778, Fisher’s Exact test for count data) than reversion in the strain containing an integrated copy of *lsr2A* at PhiC31 *attB* site (Fig. 2). This suggests that even in presence of an extra copy of *lsr2A* under the control of the constitutive kasOp* promoter [34], the deletion of the *lsr2A* native locus may have an impact on bacteria physiology or fitness.

## Discussion

In this study, we took advantage of CRISPR-Cas9 technology to design a ‘generic’ approach allowing for gene copy-specific editing. We applied it to the exploration of gene essentiality in *S. ambofaciens*. We introduced an extra copy of the target gene before attempting the deletion of the native locus. In this case, the CRISPR-Cas9-induced break must be copy-specific. One strategy could be to design a sequence of the extra copy that could not be recognized by the sgRNA. For instance, the coding sequence could be mutated to introduce synonymous mutations. However, transcriptional or post-transcriptional levels of regulation such as RNA stability, riboswitch or codon bias usage could be impacted by this kind of mutagenesis. Alternatively, a sgRNA could be designed to target specifically upstream or downstream sequences surrounding the copy of interest. In this study, we propose such an approach, the differential action of CRISPR-Cas9 system on the two gene copies being exclusively based on the difference between their genetic environments. This could be applied to the study of other repeated sequences within the genome such as multicopy genes or transposons.

One interest of our approach is that a unique CRISPR-Cas9 tool, targeting the ‘bait’ DNA, is used to edit different loci. This limits the time-consuming design and cloning of several sgRNA expression cassettes. By always targeting the same sequence regardless of the gene that is edited, this approach minimizes biases associated to differences in CRISPR-Cas9 efficiency and/or toxicity depending on the sgRNA used. This allows for the direct comparison of the editing efficiencies between different genes. A similar approach was used to efficiently edit *S. coelicolor* genome by introducing an I-*Sce*I site by homologous recombination at the vicinity of the target locus [19]. The use of CRISPR-Cas9-based approach does not require the cloning of the endonuclease recognition site within the bait DNA, and offers the additional opportunity to choose the target sequence. Notably, the risk of off-targets effects can be further reduced by choosing ‘bait’ DNA presenting a GC content that differ significantly from the host genome. For instance, in our case, the sgRNA sequence presented a GC content (40% GC) greatly lower than that of *S. ambofaciens* genome (circa 72% GC).

Depending on the sequence present between upstream and downstream regions within ‘bait’ DNA (represented by a star in Fig. 1), this CRISPR-Cas9 approach can be potentially applied to a variety of genome editing such as gene deletion, tagging, reporter gene insertion, etc. [4, 8]. The introduction of a selection marker between the upstream and downstream sequences could be used to select the deleted mutants. In this study we use a template to perform scarless deletion of two paralogs in *S. ambofaciens*. This allows the further editing of other loci within the resulting deleted clones using always the same CRISPR tool and selection marker.

In the case of scarless deletion, there is no positive selection of the deletion event, the HDR either leading to reversion or deletion. Of note the ‘revertant’ clones obtained after CRISPR-Cas9 induced-second recombination event could be used as controls of the possible toxicity of the experimental schema. Moreover, this approach gives access to the relative proportion of reversion vs. deletion obtained after targeting a gene of interest. This offers the possibility to study the impact of its mutation on bacterial fitness. Since *S. ambofaciens* is a multicellular bacteria with a complex developmental cycle [35, 36], mutations having an impact on sporulation, germination or chromosome segregation can modulate the proportion of reversion vs. deletion observed after this editing process. Indeed, in this study we observed that *lsr2A* could be deleted only in the presence of an extra copy of the gene integrated at the PhiC31 *att*B site. Furthermore, the deletion of *lsr2A* in the presence of an extra copy under the control of the synthetic promoter kasOp* is slightly less frequent than the reversal to the WT genotype (Fig. 2). This suggests that the natural level of *lsr2A* expression could confer a selective advantage to the revertant clones. Altogether our results suggest that *lsr2A* and *lsr2B* paralogs which are ubiquitous in *Streptomyces* could have distinct functions, *lsr2A* possibly being a candidate-essential gene in *S. ambofaciens* as described for *lsr2* in *M. tuberculosis* [23–25]. *S. ambofaciens lsr2* paralogs share 60.5% nucleotide sequence identity and encode proteins which are 48.6% identical and 60.4% similar (determined using EMBOSS Stretcher program). Notably Lsr2A N-terminal region contains an RGR motif that is closer to the xenogeneic silencers DNA binding motif consensus ([TS]-X-[R]-G-R-X-P-A) [22] than the corresponding Lsr2B sequence. This may confer different functionalities to Lsr2A and Lsr2B paralogs.

## Conclusion

In conclusion we have developed a CRISPR-Cas9 based technology to specifically edit one copy of a multicopy gene. This approach is based on the introduction by homologous recombination of ‘bait’ DNA at the vicinity of the target gene. We designed a single generic CRISPR-Cas9 toolkit targeting the ‘bait’ DNA that can be used to systematically edit different genes. This opens new insights on the use of CRISPR-Cas9 technology in bacteria.

## Methods

### Strains, plasmids, culture conditions and clones analysis

The strains and plasmids used in this study are listed in Additional file 1: Table S1, except intermediate constructs which are mentioned only in the text below. All the primers used in this study are presented in Additional file 2: Table S2.

*S. ambofaciens* ATCC 23877 strains were grown on solid soy flour-mannitol (SFM) medium [37] at 28 °C unless otherwise indicated. *Escherichia coli* strains were grown at 37 °C in Luria-Bertani (LB) medium. Conjugation between *S. ambofaciens* and *E. coli* ET12567/pUZ8002 containing the plasmid of interest was performed on SFM MgCl_2_ (10 mM) as previously described [37]. The plates were incubated for 5–7 days, or until conjugates became visible. Appropriate antibiotics were added to the *S. ambofaciens* growth media when needed at the following concentrations: apramycin, 50 μg/ml; kanamycin, 25 μg/ml; hygromycin 50 μg/ml; nalidixic acid, 50 μg/ml and thiostrepton, 1 μg/ml. The antibiotic concentrations used to select *E. coli* strains were the following: apramycin, 75 μg/ml; kanamycin, 25 μg/ml and hygromycin 150 μg/ml.

The *S. ambofaciens* strains were successively submitted to two or three rounds of plasmid transfer by conjugation. Firstly pSET152 plasmids harboring or lacking one *lsr2* paralog were introduced into the WT strain. Exconjugants were grown on apramycin to select clones that have integrated the plasmid at the PhiC31 *att*B site within their genome. Of note, these trans-complementation assays could also have been performed using replicative plasmids. Secondly pOSV400 derivates containing upstream and downstream regions neighboring the gene of interest were introduced within the resulting strains or OSC2. Selection was performed on hygromycin plus nalidixic acid. Finally pCRISPR-Cas9-K-sgH was introduced within the resulting clones, selection being achieved on kanamycin plus nalidixic acid. In these experiments, the nalidixic acid was used to counter-select the *E. coli* donor strain. After this last conjugation stage, exconjugants were plate on SFM supplemented with kanamycin and thiostrepton to induce the expression of Cas9. After 5 days, the clones were grown in absence of antibiotic pressure for another 2 days at 39 °C, in order to stimulate the loss of pCRISPR-Cas9-K-sgH. Thereafter, the analysis of sensitivity / resistance to kanamycin or hygromycin of at least 30 clones was performed. The genetic organization of hygromycin- and kanamycin-sensitive clones was analyzed by PCR with couple of primers located upstream and downstream the gene of interest allowing the distinction between the absence or presence of this gene sequence within the genome. For selected deleted clones of each gene of interest, the sequence of the PCR product was checked by sequencing.

### Design of a sgRNA targeting ‘bait’ DNA

The concatenated sequences of the ‘bait’ DNA (here, the hygromycin resistance encoding gene) and *S. ambofaciens* ATCC 23877 genome was submitted to CRISPRy-web site analysis (https://crispy.secondarymetabolites.org/#/input) [30] to design a N20 target sequence against the ‘bait’ DNA. This procedure allows simultaneously to scan for possible on-target sgRNA sequences and to review for off-target sites. A N20 sequence (AATACGGTCGAGAAGTAACA) among the best ranked and without identified off target (with no or 1 bp mismatch) and only 2 off-targets with 2 bp mismatches in *S. ambofaciens* ATCC 23877 genome was selected. The absence of off-target was double-checked using Cas-OFFinder (http://www.rgenome.net/cas-offinder/) [31].

### Vector cloning

For recombinant DNA manipulation, standard techniques were used. Except otherwise indicated the PCR products were subcloned into pGEM-T-easy vector (Promega™) before digestion by restriction enzymes. All insertions were checked by plasmid sequencing.

The plasmid pEX-A2 harboring an *lsr2A* synthetic expression module corresponding to *lsr2A* gene (SAM23877-RS19855) fused to hemagglutinin tag under the control of the constitutive promoter *kasO*p* [34] and the RBS of phage PhiC31 capsid gene [34] was obtained from Eurofins Genomics (see the complete sequence of the *lsr2A* expression module in Additional file 3: Table S3). The restriction sites present in this expression cassette were designed to allow a modulary sub-cloning of coding sequences of interest in frame or not with HA tag. The *Eco*RV-*Pvu*II insert (corresponding to the complete synthetic *lsr2A-ha* expression cassette) was introduced at *Pvu*II site in pSET152 vector, generating pSET152-*lsr2A-ha* construct. To remove the HA tag and generate pSET152-*lrs2A* construct, this latter plasmid was used as a template for PCR amplification performed with SBM27 and SBM28 primers followed by purification, phosphorylation and autoligation. The pSET152-*lsr2B* plasmid was obtained by amplifying *S. ambofaciens* WT genome with SBM29 and SBM31 primers, followed by digestion with *Nde*I and *Spe*I and introduction between *Nde*I and *Spe*I sites within pSET152-*lsr2A-ha*.

The upstream and downstream regions neighboring *lsr2A* gene (SAM23877-RS19855) were obtained by amplifying *S. ambofaciens* WT genome with SBM43 and SBM44 couple of primers, and SBM45 and SBM46 couple of primers, respectively. The upstream and downstream regions neighboring *lsr2B* gene (SAM23877-RS17060) were obtained by amplifying *S. ambofaciens* WT genome with SBM47 and SBM48 couple of primers, and SBM49 and SBM50 couple of primers, respectively. These regions (about 2 kb) were digested by appropriate enzymes and introduced simultaneously between *HindIII* and *SpeI* sites within pOSV400 plasmid.

The kanamycin resistance gene was obtained by amplifying with SN-MIG28 and SN-MIG29 primers the pOSV404 construct (a derivate of pSET152 that contains the gene encoding the aminoglycoside phosphotransferase from Tn5 instead of the apramycin resistance gene - lab collection). The resulting amplicon was introduced into the *Stu*I site within pCRISPR-Cas9 plasmid, generating pCRISPR-Cas9-K vector. This latter plasmid therefore contains 3 resistance markers (resistance to apramycin, thiostrepton or kanamycin) what allows a wide choice of the selection conditions depending on the possible resistance genes already present in the strain of interest.

The sgRNA targeting the ‘bait’ DNA (*hph* gene encoding the resistance to hygromycin) was constructed by fusing a crRNA and an associated tracrRNA as previously described [11]. Briefly the 20-nucleotide target sequence (AATACGGTCGAGAAGTAACA) within the sgRNA scaffold was designed to be inserted into the *Nco*I and *Sna*BI restriction sites after PCR amplification performed with SBM67 and SBM158 primers using pCRISPR-Cas9 plasmid as a template. The PCR product was directly digested, and introduced between *Nco*I and *Sna*BI sites within pCRISPR-Cas9-K.

### Statistical procedure

At least 30 exconjugants were analyzed for each strain. Data were analyzed with R software [38] and statistical significance was assessed by means of Fisher’s Exact Test for Count Data. This is a statistical significance test used in the analysis of contingency tables. This test is better adapted than the classical Chi-2 test when samples sizes are rather small and frequencies close to 100 or 0% (as in the case of the study of essential gene deletion in absence of a trans-complementing copy).

## Additional files

Additional file 1: **Table S1.** Strains and plasmids used in this study. (DOCX 57 kb)

Additional file 2: **Table S2.** Primers used in this study. (DOCX 43 kb)

Additional file 3: **Table S3.** Sequence of the synthetic *lsr2A* expression module. (DOCX 40 kb)

## Abbreviations

CRISPR/Cas: Clustered regularly interspaced short palindromic repeat and CRISPR-associated nucleases
crRNA: CRISPR RNA
HDR: Homology-directed repair
LB: Luria-Bertani medium
NAPs: Nucleoid-associated proteins
NHEJ: Non-homologous end-joining
PCR: Polymerase chain reaction
RBS: Ribosome binding site
SFM: Soy flour-mannitol medium
sgRNA: single guide RNA
tracrRNA: trans-activating CRISPR RNA
XS: Xenogeneic silencers
