# Supplementary material for: Design of a generic CRISPR-Cas9 approach using the same sgRNA to perform gene editing at distinct loci
Source: BMC Biotechnol. 2019 Mar 20;19:18. doi: 10.1186/s12896-019-0509-7 (PMC6425556; doi:10.1186/s12896-019-0509-7)
Supplement: Supplementary file 1 — Table S1. Strains and plasmids used in this study. (DOCX 57 kb) [file 12896_2019_509_MOESM1_ESM.docx]

**Table S1: Strains and plasmids used in this study**

| **Strain or plasmid** | **Relevant property(ies)*** | **Reference** |
| --- | --- | --- |
| *S. ambofaciens* strains | | |
| OSC2 alias “WT**”** | Derivative *of S. ambofaciens* ATCC 23877 devoid of the pSAM2 integrative plasmid | [1] |
| WT *att*PhiC31ΩpSET152 | OSC2 containing the plasmid pSET152 integrated at PhiC31 *att*B located between positions 4,110,159 to 4,110,196 (within SAM23877_RS18305) in *S. ambofaciens* genome | This study |
| WT *att*PhiC31ΩpSET152-*lsr2A* | OSC2 containing the plasmid pSET152-*lsr2A* integrated at PhiC31 *att*B | This study |
| WT *att*PhiC31ΩpSET152-*lsr2B* | OSC2 containing the plasmid pSET152-*lsr2B* integrated at PhiC31 *att*B | This study |
| *E. coli* strains | | |
| DH5α | General cloning strain | [2] |
| ET12567 pUZ8002 | Strain used for conjugation between *E. coli* and *Streptomyces* | [3] |
| Plasmids | | |
| pSET152 | ColE1*, oriT, attP*, *int, aac(3)-IV*  This plasmid is replicative in *E. coli* but integrative in *S. ambofaciens*. | [4] |
| pSET152-*lsr2A* | pSET152 harboring *lsr2A* gene (SAM23877-RS19855) under the control of the constitutive promoter *kasO*p* [5] and the RBS of phage PhiC31 capside gene [5] | This study |
| pSET152-*lsr2B* | pSET152 harboring *lsr2B* gene (SAM23877-RS17060) under the control of the constitutive promoter *kasO*p* [5] and the RBS of phage PhiC31 capside gene [5] | This study |
| pOSV400 | ColE1*, oriT, lacZ’*, *hph*  This plasmid is replicative in *E. coli* but not in *S. ambofaciens*. | [6] |
| pOSV400-UD-*lsr2A* | pOSV400 containing upstream (1.8 kb) and downstream regions of *lsr2A* gene (2.4 kb) (replacing *lacZ’* within the parental vector) | This study |
| pOSV400-UD-*lsr2B* | pOSV400 containing upstream (2.3 kb) and downstream regions of *lsr2B* gene (2 kb) (replacing *lacZ’* within the parental vector) | This study |
| pCRISPR-Cas9 | *oriT, aac(3)-IV,* Thio^R^*, cas9* under the P*_tipA_* promoter, sgRNA cassette under the P_ermE*_ promoter, *ori* and *rep* from the thermosensitive pSG5 replicon | [7] |
| pCRISPR-Cas9-K-sgH | pCRISPR-Cas9 containing Kana^R^ and a sgRNA targeting *hph* sequence | This study |

* *aac(3)-IV*, apramycin resistance gene; *attP*, PhiC31 phage attachment site; ColE1, origin of replication in *E. coli* (not functional in *S. ambofaciens*); *hph* : gene encoding the resistance to hygromycin; *int*, PhiC31 phage integrase gene; *lacZ’*, gene encoding the LacZα; *oriT*, origin of transfer; P*_tipA_*, thiostrepton inducible promoter; RBS, ribosome binding site; Thio^R^, resistance to thiostrepton.

**References**

1. Raynal A, Karray F, Tuphile K, Darbon-Rongere E, Pernodet JL: **Excisable cassettes: new tools for functional analysis of Streptomyces genomes**. *Appl Environ Microbiol* 2006, **72**(7):4839-4844.

2. Hanahan D: **Studies on transformation of Escherichia coli with plasmids**. *J Mol Biol* 1983, **166**(4):557-580.

3. Gust B, Chandra G, Jakimowicz D, Yuqing T, Bruton CJ, Chater KF: **Lambda red-mediated genetic manipulation of antibiotic-producing Streptomyces**. *Adv Appl Microbiol* 2004, **54**:107-128.

4. Bierman M, Logan R, O'Brien K, Seno ET, Rao RN, Schoner BE: **Plasmid cloning vectors for the conjugal transfer of DNA from Escherichia coli to Streptomyces spp**. *Gene* 1992, **116**(1):43-49.

5. Bai C, Zhang Y, Zhao X, Hu Y, Xiang S, Miao J, Lou C, Zhang L: **Exploiting a precise design of universal synthetic modular regulatory elements to unlock the microbial natural products in Streptomyces**. *Proc Natl Acad Sci U S A* 2015, **112**(39):12181-12186.

6. Boubakri H, Seghezzi N, Duchateau M, Gominet M, Kofronova O, Benada O, Mazodier P, Pernodet JL: **The Absence of Pupylation (Prokaryotic Ubiquitin-Like Protein Modification) Affects Morphological and Physiological Differentiation in Streptomyces coelicolor**. *J Bacteriol* 2015, **197**(21):3388-3399.

7. Tong Y, Charusanti P, Zhang L, Weber T, Lee SY: **CRISPR-Cas9 Based Engineering of Actinomycetal Genomes**. *ACS Synth Biol* 2015, **4**(9):1020-1029.
