# Supplementary material for: Design of a generic CRISPR-Cas9 approach using the same sgRNA to perform gene editing at distinct loci
Source: BMC Biotechnol. 2019 Mar 20;19:18. doi: 10.1186/s12896-019-0509-7 (PMC6425556; doi:10.1186/s12896-019-0509-7)
Supplement: Supplementary file 2 — Table S2. Primers used in this study. (DOCX 43 kb) [file 12896_2019_509_MOESM2_ESM.docx]

**Table S2: Primers used in this study**

| Primer name | Target region | Sequence (5’-3’)* |
| --- | --- | --- |
| *Primers used for vector cloning* | | |
| SBM27 | pSET152 plasmid | actagttgactaaccgcgg |
| SBM28 | *lsr2A* | TTAGCCGTTGGCCTTCTCGTACG |
| SBM29 | *lsr2B* | ccatatgGCGCAGCGTGTCGTGGTCAC |
| SBM31 | *lsr2B* | actagtTCACTGCGCCGCGGTGAACG |
| SBM43 | *lsr2A* upstream region | aagcttAGGGCAGGCACGGCGACCAC |
| SBM44 | *lsr2A* upstream region | gaattcTGTGCCACCGGATTTCCTTTCATCG |
| SBM45 | *lsr2A* downstream region | gctagcCTGATCACCCGCCCCGCACCAC |
| SBM46 | *lsr2A* downstream region | actagtATCCGGGAGCCGGCCTCGTC |
| SBM47 | *lsr2B* upstream region | aagcttCACCGCGCACATGCGCTCCT |
| SBM48 | *lsr2B* upstream region | gaattcCACGACACGCTGCGCCACGA |
| SBM48 | *lsr2B* downstream region | gctagcCGGGGCCGGATCCCGAAGAA |
| SBM50 | *lsr2B* downstream region | actagtCAGCCCACCGCCAGCAGCAC |
| SBM67 | pCRISPR-Cas9 | ACGCCTACGTAAAAAAAGCACCGACTCGGTGCC |
| SBM158 | sgRNA-HygroR, pCRISPR-Cas9 | CATGCCATGGAATACGGTCGAGAAGTAACAGTTTTAGAGCTAGAAATAGC |
| SN-MIG28 | Kana^R^ | GTTCATGTGCAGCTCCATCAG |
| SN-MIG29 | Kana^R^ | TCAGAAGAACTCGTCAAGAAGGCG |
| *Primers used for genome analysis* | | |
| SN-MIG32 | *lsr2A* upstream region | CCCGTCGACACCGATGTCTAC |
| SN-MIG33 | *lsr2A* downstream region | TGCTGGAGTGGCTGGACTG |
| SN-MIG34 | *lsr2B* upstream region | CGCGTAGCATTGTAGGAAAC |
| SN-MIG35 | *lsr2B* downstream region | GCTCCAGACTTTAGCTGATCAC |

* Restriction sites are underlined. The 20 nt sgRNA target sequence is written in bold.
