# Supplementary material for: Design of a generic CRISPR-Cas9 approach using the same sgRNA to perform gene editing at distinct loci
Source: BMC Biotechnol. 2019 Mar 20;19:18. doi: 10.1186/s12896-019-0509-7 (PMC6425556; doi:10.1186/s12896-019-0509-7)
Supplement: Supplementary file 3 — Table S3. Sequence of the synthetic lsr2A expression module. (DOCX 40 kb) [file 12896_2019_509_MOESM3_ESM.docx]

**Table S3: Synthetic *lsr2A* expression module**

| **Feature positions** | **Sequence (5’-3’)** |
| --- | --- |
| 1-6: *Eco*RV restriction site (underlined)  7-68: *kasO*p* promoter (with -35, -10 sequences in light blue)  69: Predicted transcription start site (bold A)  91-96: RBS (ribosome binding site, written in pink)  101-106: *Nde*I restriction site (underlined) and start codon (bold ATG)  437-445: *Not*I restriction site (underlined)  446-472: HA tag (written in orange)  473-478: Stop codons (in bold)  479-484: *Spe*I restriction site (underlined)  484-491: Stop codons (in bold)  492-497: *Sac*II restriction site (underlined) | gatatctgttcacattcgaaccgtctctgctttgacaacatgctgtgcggtgttgtaaagtcgtggcc**A**ggagaatacgacagtctaagtaaggagtgtccat**atg**GCACAGAAGGTTCAGGTCCTTCTTGTCGACGACCTCGACGGTGGCGAGGCAGACGAGACCGTGACGTTCGCGCTGGACGGCAAGACGTACGAGATCGATCTCACCACCGCCAATGCGGACAAGCTCCGTGGACTTCTCGACCCGTACGTGAAGGGTGGTCGTCGCACCGGGGGCCGCGCCTCGGGCGGACGCGGCAAGGCGCGCGCCTCCGCCGGCGGCAGCCAGGACACCGCGGCGATCCGCGCCTGGGCCAAGGAGAACGGCTACGACGTCAACGACCGCGGTCGTGTCCCCGCGATGATCCGCGAGGCGTACGAGAAGGCCAACGGCgcggccgcgTACCCCTACGACGTGCCGGACTACGCG**tgataa**actagt**tga**c**taa**ccgcgg |
